# Effect of Eicosapentaenoic Acid Supplementation on Murine Preadipocytes 3T3-L1 Cells Activated with Lipopolysaccharide and/or Tumor Necrosis Factor-α

**DOI:** 10.3390/life11090977

**Published:** 2021-09-16

**Authors:** Anna Zając-Grabiec, Karoline Bartusek, Katarzyna Sroczyńska, Tadeusz Librowski, Joanna Gdula-Argasińska

**Affiliations:** 1 Department of Radioligands, Faculty of Pharmacy, Medical College, Jagiellonian University, Medyczna 9, 30-688 Kraków, Poland; anna25.zajac@student.uj.edu.pl (A.Z.-G.); katarzyna91kardas@student.uj.edu.pl (K.S.); mflibrow@cyf-kr.edu.pl (T.L.); 2 Faculty of Pharmacy, Rheinische Friedrich-Wilhelms University of Bonn, An der Immenburg 4, 53121 Bonn, Germany; karolinebartusek@yahoo.de

**Keywords:** 3T3-L1 cells, adipocytes, eicosapentaenoic acid, inflammation, LPS, TNF-α

## Abstract

The beneficial effect of n-3 fatty acids can be related to anti-inflammatory properties. The aim of the study was to analyzed the effect of eicosapentaenoic acid (EPA) on 3T3-L1 cells (murine embryonic fibroblasts‒preadipocytes) activated with inflammatory factors (IF). Cells were incubated with 50 µmol of EPA for 48 h, and then activated with lipopolysaccharide (LPS) or tumor necrosis factor-α (TNF-α). The level of cycloxygenase-2 (Prostaglandin-Endoperoxide Synthase 2, PTGS2, COX-2), cytosolic prostaglandin synthase E2 (cPGES), fatty acid binding protein 4 (FABP4), toll-like receptor 4 (TLR4), glucose receptor type 4 (GLUT-4), and cannabinoid receptor 2 (CB2) was determined using Western blot analysis. The phospholipase A2 (Pla2g4a), and prostaglandin-Endoperoxide Synthase 2 (Ptgs2) gene expression was analyzed by real-time qPCR. After EPA and IF activation, a significant decrease in the COX-2, cPGES, and TRL4 protein levels was observed. Incubation of cells with EPA and IF resulted in a decrease in Ptgs2 and an increase in the Pla2g4a gene. A significant increase in the CB2 protein was observed in adipocytes co-treated with EPA and IF. The results indicated an anti-inflammatory properties of EPA. Interestingly, the activation of the GLUT4 receptor by EPA suggests an unique role of this FA in the regulation of the adipocyte metabolism and prevention of insulin resistance.

## 1. Introduction

Adipose tissue has insulating, shock-absorbing, and above all, energy storage functions. It also acts as an endocrine organ. Adipose tissue stores energy in the form of triacylglycerols in the lipogenesis process, but also regulates energy mobilization in the process of lipolysis [1,2,3]. De novo adipocyte differentiation in response to excess energy supply acts as an adaptive mechanism by increasing the size of adipose tissue and affects the proper functions of adipocytes [1,2,3,4]. This adaptive mechanism prevents systemic lipid overload, which is the leading cause of insulin resistance. Adipose tissue has a remarkable ability to rebuild and adapt to the nutritional state of the body. Adipocytes undergo changes during their life and are very dynamic tissue [1,2,3,4]. One of the major regulators of adipogenesis is peroxisome proliferator-activated receptor *γ* (PPARγ), which is responsible for the differentiation and phenotype of adipocytes [2,3].

A positive energy balance, associated with an excessive supply of nutrients and a lack of physical activity, leads to excessive accumulation of fat in adipocytes and induces inflammation [1]. It is presumed that the stress caused by anabolic processes related to overnutrition initiates catabolic processes, including the inflammatory response and oxidative stress, to maintain homeostasis [2].

If too much energy is supplied, the adipose tissue may grow as hypertrophy and hyperplasia [1,2,3,4]. A positive energy balance causes an increased synthesis of acetyl-CoA and nicotinamide adenine dinucleotide (NADP) in mitochondria, which in turn causes an increase in the biosynthesis of reactive oxygen species (ROS), damaging cell organelles [1,2,3,4,5,6,7]. Cells, in defending themselves against the effects of ROS, block the flow of energy substrates (especially glucose) by reducing the number of insulin receptors, as well as an insulin receptor substrates (IRS-1) type-4 glucose transporter (GLUT-4) which interferes with the proper functioning of insulin [2,3,8,9,10,11,12,13]. Pro-inflammatory cytokines released from macrophages or directly from adipocytes include tumor necrosis factor-α (TNF-α), interleukins (IL-1β, IL-6), and a C-reactive protein (CRP). Cytokines released from visceral adipose tissue induce low-level systemic inflammation and promote insulin resistance. TNF-α inhibits triacylglycerol synthesis by suppressing PPARγ and GLUT4. In addition, TNFα inhibits the insulin-dependent lipolysis process, increases the cyclic adenosine monophosphate (cAMP) pool, and reduces the amount of perilipin protein, resulting in increased release of free fatty acids (FFA) from adipose tissue [11,12,13].

Chronic inflammation induction due to endocrine activity of adipose tissue affects the molecular mechanisms of insulin action and also contributes to the development of other metabolic complications, often accompanying diabetes [11]. The pro-inflammatory pathways of kinase-κβ/nuclear factor κB (IKK-β/NF-κB) and the N-terminal protein kinase c-Jun (JNK1), both with increased activity, are observed in obese people, and are responsible for the disruption of signaling through the insulin receptors [2,11]. The adipose tissue inflammation results in further complications, such as insulin and catecholamine resistance as well as lipotoxicity, and is associated with an increased incidence of type 2 diabetes, insulin resistance, atherosclerosis, hyperlipidemia, heart disease, and high blood pressure [1,2,3,4]. Increased release of FFA from an intensive lipolysis process resulted in their uptake by cells, mainly hepatocytes and myocytes [2]. Visceral tissue has a greater number of β-adrenergic receptors and shows their increased expression [12]. This translates into increased lipolytic activity, which in turn increases the level of FFA in the blood [12].

Factors influencing the development of obesity include changes in the composition of the microflora and increased lipopolysaccharide (LPS) concentrations, which then leads to an increase in intestinal permeability and chronic low-grade inflammation, as well as an increase in energy intake with a simultaneous reduction in energy expenditure [5,6,7]. The gut-brain axis is a complex neurohumoral communication network necessary for the maintenance of metabolic homeostasis and is a potential target of personalized metabolic therapy [5,6,7]. FFA propagate inflammation by binding to the toll-like TLR4 and TLR2 receptors and activate the pro-inflammatory NF-κB pathway [1,2,3,4,13]. Activation of NF-κB increases the synthesis and secretion of cytokines and chemokines such as monocyte chemoattractant protein (MCP1) by adipocytes, leading to infiltration of macrophages to the adipose tissue [11,13]. Overexpression of TLR2 and TLR4 is increased in the adipose tissue of obese individuals, indicating that these receptors are involved in obesity-related inflammatory signaling [11]. Other potential FFA sensors include fatty acid-binding protein 4 (FABP4), which can promote pro-inflammatory signaling in macrophages [11].

Fatty acids from the n-3 family, such as eicosapentaenoic acid (EPA), have anti-inflammatory properties. These fatty acids, as peroxisome proliferator-activated receptor (PPAR) ligands, may modulate the immune response and inhibit the expression of inflammatory genes [9,10,11,13].

The murine 3T3-L1 cell line model was the first and the best characterized in the adipogenesis research studies and, therefore, is commonly used in the biochemistry of adipocytes [14,15].

The aim of the study was to evaluate the pro- or anti-inflammatory effect of eicosapentaenoic acid (EPA, C20:5, n-3) on pro-inflammatory genes on the mRNA or protein level in murine preadipocytes 3T3-L1 activated with LPS and TNF-α.

## 2. Materials and Methods

### 2.1. Cell Cultures

Mus musculus embryonic fibroblast cells (3T3-L1, CL-173, ATCC, Manassas, VA, USA) were cultured in DMEM medium with 10% Fetal Bovine Serum (ATCC) and with streptomycin (100 µg/mL) and penicillin (100 IU/mL) (ATCC). Cells were maintained at 37 °C in a humidified atmosphere which contained 5% CO_2_ and seeded to 75 cm^2^ Falcon flasks (Sarsted, Nümbrecht, Germany) at a density of 5 × 10^5^ cells in 12 mL of medium. During every step of the procedure, cell morphology was investigated by an inverted light microscope (Olympus, Tokyo, Japan). Cell viability during culturing was assessed using the Trypan Blue Exclusion Test.

### 2.2. T3-L1 Cells Differentiation

The 3T3-L1 cells were seeded in 60-mm dishes (Sarstedt) at a density of 5 × 10^5^ cells in basal medium-BM I (DMEM high glucose, 10% Newborn Calf Serum NCS, antibiotics) for 0–3 days (48 h confluence), as proposed in the protocol from Zebisch et al., (2012). After this time, the differentiation medium (BM I, isobutyl-1-methylxanthine IBMX 0.5 mol, insulin 1 µg/mL, dexametason 0.25 µmol/mL, rosiglitazone 2 µmol/mL) was used for the next 3–5 days (Merck, Darmstadt, Germany). After this time, differentiation medium II was used for the next 5–7 days (BM I, insulin 1 µg/mL), and the basal medium was used for mature adipocytes for 7–14 days. The differentiation process was easily visible. Intracellular lipid droplets appeared at around 7 days and increased in both number and size over the following days. The adipocytes after differentiation were used for the experiment.

The 3T3-L1 cells were incubated with 50 µmol of eicosapentaenoic acid (EPA, dissolved in ethanol) for 48 h and then activated with 10 ng/µL of lipopolysaccharide (LPS isolated from *E. coli*) or tumor necrosis factor-α (TNF-α, human recombinant) (Sigma-Aldrich, Saint Louis, MO, USA). All experiments were carried out in triplicate, and the number of samples for each group was five (N = 5).

### 2.3. ApoTox-Glo Triplex Assay

The ApoTox-Glo triplex assay (Promega, Madison, WI, USA) was performed to assess the cytotoxicity of the treatment compounds, as well as the cell viability and apoptosis, as per manufacturer instruction.

### 2.4. Western Blot

After removing the media, 2 mL of ice-cold DPBS (Dulbecco’s Phosphate-Buffered Saline) was added to the plates to clear any residual phenol red medium that could interfere with the colorimetric measurement of total protein concentration.

The cells were scrapped, transferred to Eppendorf tubes, and centrifuged at 1200× *g* for 7 min at 4 °C. The supernatants were removed. One hundred µL of M-PER (Thermo Fisher Scientific, Rockford, IL, USA), 1 µL of protein inhibitors cocktail (Merck), and 1 µL of phosphatase Inhibitors (Cayman Chemicals, Ann Arbor, MI, USA) was added to each sample. They were then centrifuged at 10,000× *g* for 10 min at 4 °C. A part of the supernatant was taken to measure the concentration of total proteins based on the use of the Coomassie Brillant Blue G-250 Bradford reagent, according to the instruction (Bio-Rad, Hercules, CA, USA). The remaining supernatant was mixed at 1:1 with the sample Laemmli Buffer with 2-mercaptoethanol (Bio-Rad) and warmed for 10 min at 85 °C.

Ten percent SDS polyacrylamide gel electrophoresis was used for protein separation (110 V per 90 min, running buffer: Tris–HCl + glycine + SDS, Bio-Rad). After this, the electrophoresis proteins were transferred to PVDF membranes (100 V at 4 °C per 1 h, blotting buffer: Tris–HCl + glycine + methanol, Bio-Rad). One percent of casein solution in the TBS buffer (Bio-Rad) was used as a blocking agent. After washing with TBS-Tween buffer (Bio-Rad), membranes were incubated with primary antibodies (overnight, at 4 °C). Antibodies against cyclooxygenase 2 (COX-2, 1:1000, Thermo Fisher Scientific), cytosolic prostaglandin synthase E2 (cPGES, 1:1000, Cayman Chemicals), fatty acid-binding protein 4 (FABP4, 1:500, Thermo Fisher Scientific), glucose transporter 4 (GLUT4, 1:500, Sigma-Aldrich), cannabinoid receptor 2 (CB2, 1:500, Cell Signaling, Beverly, MA, USA), toll-like receptor 4 (TLR4 1:1000, Thermo Fisher Scientific) and β-actin, as an endogenous control (1:1000; Thermo Fisher Scientific), were used. The secondary antibody was anti-rabbit IgG-HRP (1:2000; 1.5 h, at room temperature) (Thermo Fisher Scientific). Chemiluminescence detection of the immunoblot was detected using Clarity Western ECL Luminol Substrate (Bio-Rad). Chemi Doc Camera with Image Lab software (Bio-Rad) was used for the quantification of the signal optical density. Results were calculated as a ratio of the optical density of protein of interest to the optical density of β-actin, and expressed as fold changes of control. Original Western blot figures are provided in Supplementary Materials.

### 2.5. Real-Time qPCR

After the experiments, 3T3-L1 cells were collected with a scrapper in the presence of an RNALater (Thermo Fisher Scientific) and stored at −20 °C for further studies. Total RNA was isolated using an PureLink RNA isolation kit, according to the manufacturer instruction (Thermo Fisher Scientific). RNA concentration was normalized to 20 ng/μL. Reverse transcription was performed with a High-Capacity Reverse Transcription Kit (Thermo Fisher Scientific). A qPCR 96-wells fast reaction plate was performed with TaqMan Master mix and TaqMan gene assays for Ptgs2 (Mm00478374_m1), Pla2g4a (Mm00447040_m1), and Gapdh (Mm99999915_g1), as an endogenous control, according to the manufacturer’s protocol on Applied Biosystems 7500 Fast Real-Time PCR Instrument (Applied Biosystems, Foster City, CA, USA). The ΔΔCq method was used to calculate relative expression.

### 2.6. Statistic

For each analyzed effects from inflammatory factors and EPA treatment, the data were obtained as triplicate measurements average from two independent experiments. Values are presented as means ± SD. Two-way ANOVA was conducted to evaluate the effects from EPA and LPS and/or TNF-α treatment. Protein expression and activity were assumed as dependent variables, and inflammatory factors activation or EPA supplementation were exploratory variables. It tested three null hypothesis: H1—the means of the dependent variable were equal for different values of EPA; H2—the means of the dependent variable were equal for different values of inflammatory factors; and H3—there was no interaction. Calculations were completed using STATISTICA 13.1 (StatSoft Sp. z o.o., Kraków, Poland) and statistical significance was established as *p* ≤ 0.05.

## 3. Results

### 3.1. Cells Viability

No cytotoxic effects or apoptosis were observed in the 3T3 cells treated with 50 µmol of EPA for 24 h and after incubation with inflammatory factors. Cell viability varied from 99% to 100%.

### 3.2. Protein Levels

**Figure 1:**
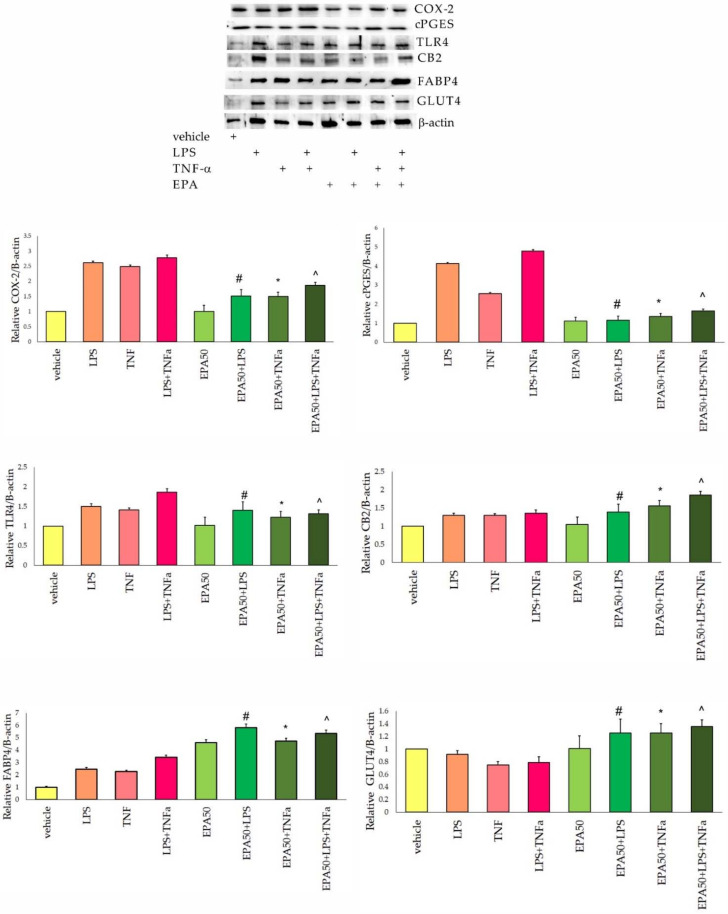
Relative cyclooxygenase 2 (COX-2), cytosolic prostaglandin E2 synthase (cPGES), toll-like receptor 4 (TLR4), cannabinoid receptor 2 (CB2), fatty acid binding protein 4 (FABP4), and glucose transporter 4 (GLUT4) protein level in 3T3-L1 cells incubated with eicosapentaenoic acid (EPA) and activated with lipopolysaccharide (LPS) and tumor necrosis factor α (TNF-α). Results were normalized to β-actin. #-vs. LPS, *p* < 0.05, *-vs. TNF-α, *p* < 0.05, ^-vs. LPS+TNF-α, *p* < 0.05. N = 5.

The cyclooxygenase 2 level was over two times higher in 3T3-L1 cells activated with inflammatory factors (IF), when compared to the vehicle group. EPA supplementation resulted in a significant decrease in this protein (Figure 1). Statistical differences for COX-2 expression in 3T3-L1 cells were observed for inflammatory factors (IF) treatment (F(2,35) = 39.9, *p* = 0.000), for EPA supplementation (F(2,35) = 22.1, *p* = 0.000), and for IF–EPA interaction (F(2,35) = 43.7, *p* = 0.000). The highest level of the cPGES protein was observed in adipocytes activated with LPS and TNF-α. In 3T3-L1 cells after incubation with EPA, a significant decrease in cPGES was observed (Figure 1). For the cPGES protein level, statistical differences were noted after IF activation (F(2,35) = 27.6, *p* = 0.000), after EPA supplementation (F(2,35) = 35.9, *p* = 0.000) and for IF–EPA interactions (F(2,35) = 45.6, *p* = 0.000). The level of CB2 receptor increased slightly in adipocytes incubated with EPA and activated with IF. Significantly higher was noted in the EPA+LPS+TNF-α group (Figure 1). For the CB2 protein, statistical differences were noted only after EPA supplementation (F(2,35) = 13.1, *p* = 0.000). The high values of TLR4 protein were observed in adipocytes after IF activation. The highest level was noted in LPS+TNF-α-treated cells. Supplementation with EPA resulted in a significant decrease in this protein (Figure 1). TLR4 expression differed statistically in cells after IF treatment (F(2,35) = 16.8, *p* = 0.000), after EPA supplementation (F(2,35) = 15.2, *p* = 0.001) and for IF–EPA interaction (F(2,35) = 16.0, *p* = 0.001). For the FABP4 protein, a significant increase was observed in 3T3-L1 cells activated with IF when compared to the vehicle. After EPA incubation and IF treatment, an increase in FABP4 was noted (Figure 1). The expression of FABP4 differed significantly in 3T3 cells after EPA supplementation (F(2,35) = 15.7, *p* = 0.000), after IF treatment (F(2,35) = 23.4, *p* = 0.000), and for IF–EPA interaction (F(2,35) = 10.7, *p* = 0.000). The GLUT4 level decreased significantly after TNF-α and LPS+TNF-α treatment. In adipocytes incubated with EPA, a significant increase in this protein was noted (Figure 1). For the GLUT4, statistical differences were noted after EPA treatment (F(2,35) = 45.0, *p* = 0.000), and for IF–EPA interactions (F(2,35) = 32.0, *p* = 0.000).

### 3.3. Ptgs2 and Pla2g4a Gene Expression

**Figure 2:**
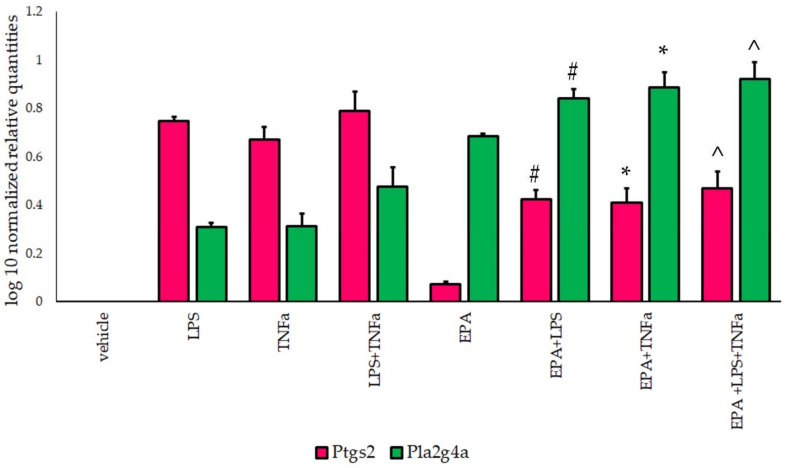
Relative prostaglandin-endoperoxide Synthase 2 (Ptgs2) and phospholipase A2 (Pla2g4a) gene expression (presented as log10), normalized to Gapdh, in 3T3-L1 cells incubated with eicosapentaenoic acid (EPA) and activated with lipopolysaccharide (LPS) and tumor necrosis factor α (TNF-α). #-vs. LPS, *p* < 0.05, *-vs. TNF-α, *p* < 0.05, ^-vs. LPS+TNF-α, *p* < 0.05. N = 5.

For Ptgs2, gene overexpression was observed in 3T3-L1 cells activated with IF. The highest value was noted in the LPS+TNF-α group. After incubation with EPA, a significant increase in the Ptgs2 gene was observed (Figure 2). Statistical differences were noted after EPA supplementation (F(2,35) = 52.0, *p* = 0.000), after IF treatment (F(2,35) = 25.9, *p* = 0.000), and for IF–EPA interactions (F(2,35) = 31.0, *p* = 0.000).

An increase in the Pla2g4a gene was observed in adipocytes after IF activation. Incubation with EPA resulted in a statistically significant increase in Pla2g4a expression (Figure 2). For Pla2g4a, differences were noted for EPA supplementation (F(2,35) = 61.8, *p* = 0.000), for IF treatment (F(2,35) = 31.0, *p* = 0.000), and for IF–EPA interactions (F(2,35) = 29.2, *p* = 0.000).

## 4. Discussion

Inflammation is a physiological process that has been described since ancient times as a response to infection or trauma, with major symptoms of redness and swelling with fever and pain, and possibly loss of function. Many of the major contributors to inflammation are well known with the likes of transcription factors such as nuclear factor kappa B (NF-κB); pro-inflammatory cytokines, including interleukins (IL-1 and IL-6) and tumor necrosis factor alpha (TNF-α); products of cyclooxygenase and lipoxygenase; and lipid mediators, including prostaglandins and leukotrienes [1,2,8,9,10,11]. The current evidence suggests that the inflammation process is much more complex as many other molecules coordinate the entire process in an interrelated network. One of the latest developments is the discovery of the inflammasome, a tightly regulated intracellular multi-protein complex of the innate immune system that is at the center of signaling pathways in various inflammatory diseases, including obesity-induced inflammation, insulin resistance, and autoimmunity [10,11].

Eicosapentaenoic acid (EPA) belonging to n-3 long-chain fatty acids is a component of many parts of the body including phospholipids in cell membranes. Intake of EPA provides health benefits as it protects against cardiovascular disease and exhibits anti-inflammatory effects [16,17,18,19,20,21]. n-3 fatty acids EPA and docosahexaenoic acid (DHA) are linked to decrease inflammatory processes [8,9,10,11]. Since n-3 PUFAs play a key role in regulating body homeostasis, it is supposed to protect against inflammatory diseases and other chronic illness, such as diabetes, obesity, and neurological degeneration [22]. Resolvins and protectins generated from the n-3 PUFAs are described to facilitate the resolution of inflammation. These molecules are examples of endogenous anti-inflammatory and pro-resolving mediators which repeal the effects of the pro-inflammatory signaling system [16,17,18,19,20]. n-3 FA exhibit numerous effects on cell-like membrane fluidity, phospholipid bilayer of cell membrane, as well as signaling across the membrane. In addition, n-3 PUFA counteracts inflammation by activation of the anti-inflammatory transcription factor peroxisome proliferator-activated receptor PPARγ [16,17].

In our study, we observed that EPA alone did not affect pro-inflammatory proteins (COX-2, cPGES, TLR4, and CB2) in adipocytes. Incubation with EPA resulted in an increase in FABP4 and a slight increase in the GLUT4 protein when compared to the vehicle. EPA in In 3T3-L1 cells supplemented with EPA upregulation of Ptgs2 and Pla2g4a genes was observed. After supplementation with EPA and activation with inflammatory factors, a significant decrease in COX-2, cPGES, and TRL4 protein levels was observed in adipocytes, when compared to the groups treated with LPS and TNF-α. These results suggest the anti-inflammatory properties of EPA. Reports of recent years clearly indicate that n-3 FA and their derivatives are essential for proper growth and development; act in an immunomodulating, anti-inflammatory, anti-arteriosclerosis, anticancer manner; and are resolvents of inflammation [8,9,10,16,17,18,19]. N-3 fatty acids, mainly derived from fish oil acting competitively to n-6 FA, inhibit arachidonic acid metabolism and, thus, reduce the production of pro-inflammatory prostaglandins and leukotrienes [16,17]. EPA not only exchanges arachidonic acid in membrane phospholipids, but is also an inhibitor of cyclooxygenase [16,17,19].

The beneficial effect of n-3 fatty acids can also be related to the anti-inflammatory activity of oxidized derivatives of EPA [16,17]. Thus, the diet supplemented with n-3 FA may exhibit protective and therapeutic activity in inflamed adipose tissue. Our results confirmed this observation.

Biosynthesis of eicosanoids in mammalian cells is generally initiated by an activation of phospholipase A2 (cPLA2) and the release of arachidonic acid and other PUFAs from sn-2 positon of cell membrane phospholipids [8,9,10]. In our study, incubation of EPA and inflammatory factors resulted in a decrease in the Ptgs2 gene and an increase in the Pla2g4a gene, which suggested the release of EPA from phospholipids of adipocyte membranes.

A significant increase in the CB2 protein was observed in adipocytes after EPA supplementation and activation with inflammatory factors. The cannabinoid CB1 and CB2 receptors belong to the G protein-coupled receptors (GPCR). The ligands for the CB1 and CB2 receptors are cannabinoids, eicosanoids, and aminoalkylindoles. CB1 is mainly found in the nervous system, while CB2 is expressed in cells of the immune system, but is also present in other peripheral organs and cell types involved in the immune response. CB2 receptors have the ability to control the activation and migration of immune cells, and represent key regulators of inflammatory responses [21,22,23]. Fatty acids can also convert directly into N-acylethanolamines (NAE). A study by Balvers et al., (2010) showed that 3T3-L1 adipocytes synthesize the DHEA and EPEA endocannabinoids from their respective precursors. The authors demonstrated that DHEA and EPEA reduced the excretion of IL-6 and MCP-1 upon stimulation of adipocyte LPS and have anti-inflammatory properties [23]. An increase in the CB2 protein in adipocytes after EPA supplementation and IF activation suggested the formation of endocannabinoids.

In our study, incubation with EPA and activation with inflammatory factors resulted in an increase in the GLUT4 protein in 3T3-L1 cells, which suggests that EPA improves glucose uptake by adipocytes under inflammatory stress. In the work of Prostek et al., (2016), 3T3-L1 cells were cultured for 48 h in the presence of 100-µM EPA or 50-µM DHA. The authors showed that DHA and EPA can induce lipolysis, exert anti-inflammatory effects, and increase insulin sensitivity in 3T3-L1 adipocytes [24].

Adipocytes actively control metabolism and are also an endocrine organ by secreting specific adipokines that have pro-inflammatory and anti-inflammatory effects. The peroxisome proliferation-activated receptor, PPAR-γ, is critical in adipogenesis as it acts as a regulator of adipocyte differentiation and function and the absorption of fatty acids. PPAR-γ is also a key regulator of inflammatory and immune responses [25,26]. Pro-inflammatory cytokines elicit changes in gene expression that may affect metabolic regulation. Treatment of adipocytes with TNF-α reduces the expression of the insulin-responsive glucose transporter GLUT4 and PPAR-γ. Pro-inflammatory cytokines and saturated fatty acids can upregulate genes involved in ceramide biosynthesis and induce inflammation and insulin resistance [26,27]. In our study, we observed the reduction in TLR4 and an increase in GLUT4 in adipocytes after EPA supplementation even with IF activation. In the study of Tishinsky et al., (2011), EPA and DHA increased the secretion of adiponectin, anti-inflammatory adipokine involved in obesity, and related diseases. EPA with combination with rosiglitazone (a PPAR-γ agonist) improved adiponectin secretion which suggested the therapeutic importance of long-chain n-3 PUFAs, alone or in combination with a PPAR-γ agonist [27]. It has also been shown that EPA and DHA, alone or combined, in the adipogenesis modulation in cultured 3T3-L1 adipocytes, affected the cell differentiation, maturation, and consequently, and reduced adipogenesis via the PPARγ-CIDEC pathway [28]. EPA has been shown to have beneficial metabolic effects in the adipocytes of brown adipose tissue by activating thermogenesis. EPA increases the expression of the thermogenesis genes and the UCP1 protein and improved the mitochondrial function of adipocytes [29,30].

Fatty acid-binding protein 4 (FABP4) has been demonstrated to be secreted from adipocytes and associated with lipolysis and inflammation [31,32,33]. In our study, we observed an increase in the FABP4 protein in adipocytes incubated with EPA and activated with inflammatory factors, which may suggest an increase in the use of the lipolysis process in inflamed adipocytes. In the study of Furuhashi et al., (2016), it was noted that incubation of 3T3-L1 cells with EPA or DHA had no effect on the short-term secretion of FABP4. Gene expression and long-term secretion of FABP4 was significantly reduced by treatment with EPA or DHA [33]. On the other hand, elevated levels of the FABP4 protein, which is a marker of adipocyte differentiation [2,14], may indicate adipocyte maturation during the course of the experiment. Certainly, further studies are needed, both at the FABP4 gene and protein level, in relation to dose-effect-time exposure to EPA in inflamed adipocytes. Limitations of our study are the murine, not the human in vitro model of inflamed adipocytes, short-time of exposure to EPA, as well as the using cells not the whole organism.

Our results suggest that EPA significantly contributes to the mitigation of adverse effects caused by inflammatory factors in the adipocytes. The data indicated the anti-inflammatory properties of EPA. Interestingly, the activation of the GLUT4 receptor by EPA suggests a unique role of this FA in the regulation of adipocyte metabolism and prevention of insulin resistance. N-3 fatty acids and their metabolites, which are ligands for PPARs and NF-ĸB transcription factors, may be used as nutraceuticals in the regulation of the immune response.

Therefore, further research on the effectiveness of eicosapentaenoic acid and its derivatives seems to be necessary, mainly in order to determine its potential and clinical applications as therapeutic agents.

## Data Availability

The original data obtained in this work are available upon request from the corresponding author.

## Supplementary Materials

The following are available online at https://www.mdpi.com/article/10.3390/life11090977/s1, Figure S1. Original Western blot figures.
