# Supplementary material for: Effect of Eicosapentaenoic Acid Supplementation on Murine Preadipocytes 3T3-L1 Cells Activated with Lipopolysaccharide and/or Tumor Necrosis Factor-α
Source: Life (Basel). 2021 Sep 16;11(9):977. doi: 10.3390/life11090977 (PMC8472223; doi:10.3390/life11090977)
Supplement: Supplementary file 1 [file life-11-00977-s001.zip › life-1347093-supplementary.pdf]

Supplementary Materials of:

Effect of Eicosapentaenoic Acid Supplementation on Murine Preadipocytes 3T3-L1 Cells Activated with Lipopolysaccharide and/or Tumor Necrosis Factor-α

Anna Zając-Grabiec [1], Karoline Bartusek [2], Katarzyna Sroczyńska [1], Tadeusz Librowski [1] and Joanna Gdula-Argasińska [1,*]

[1] Department of Radioligands, Faculty of Pharmacy, Medical College, Jagiellonian University, Medyczna 9, 30-688 Kraków, Poland; anna25.zajac@student.uj.edu.pl (A.Z.-G.); katarzyna91kardas@student.uj.edu.pl (K.S.); mflibrow@cyf-kr.edu.pl (T.L.)

[2] Faculty of Pharmacy, Rheinische Friedrich-Wilhelms University of Bonn, An der Immenburg 4, 53121 Bonn, Germany; karolinebartusek@yahoo.de

* Correspondence: jargasinska@cm-uj.krakow.pl

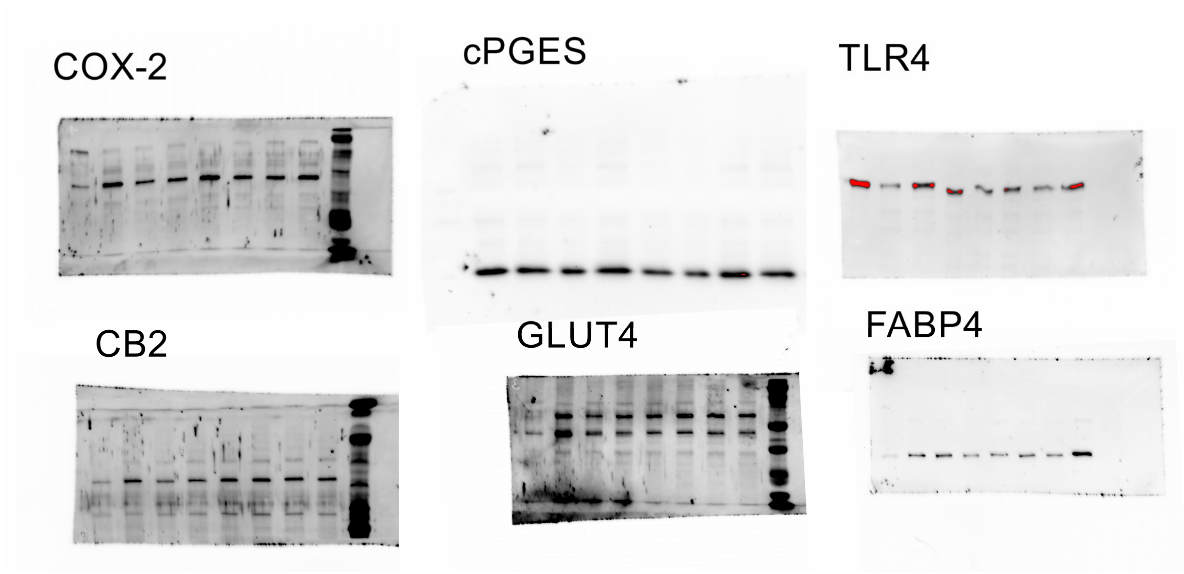

Figure S1. Original Western blot figures.
